# *MTL* genotypes, phenotypic switching, and susceptibility profiles of *Candida parapsilosis* species group compared to *Lodderomyces elongisporus*

**DOI:** 10.1371/journal.pone.0182653

**Published:** 2017-08-03

**Authors:** Aylin Döğen, Banu Metin, Macit Ilkit, G. Sybren de Hoog, Joseph Heitman

**Affiliations:** 1 Department of Pharmaceutical Microbiology, Faculty of Pharmacy, University of Mersin, Mersin, Turkey; 2 Department of Food Engineering, Faculty of Engineering and Natural Sciences, Istanbul Sabahattin Zaim University, Istanbul, Turkey; 3 Division of Mycology, Department of Microbiology, Faculty of Medicine University of Çukurova, Adana, Turkey; 4 Westerdijk Fungal Biodiversity Centre, Utrecht, the Netherlands; 5 Institute for Biodiversity and Ecosystem Dynamics, University of Amsterdam, Amsterdam, the Netherlands; 6 Department of Molecular Genetics and Microbiology, Duke University School of Medicine, Durham, North Carolina, United States of America; Louisiana State University, UNITED STATES

## Abstract

Reference isolates of *Candida parapsilosis* (n = 8), *Candida metapsilosis* (n = 6), *Candida orthopsilosis* (n = 7), and *Lodderomyces elongisporus* (n = 11) were analyzed to gain insight into their pathobiology and virulence mechanisms. Initial evaluation using BBL Chromagar Candida medium misidentified *L*. *elongisporus* isolates as *C*. *albicans*. Polymerase chain reaction analysis of isolate *MTL* idiomorphs revealed that all *C*. *parapsilosis* isolates were *MTL***a** homozygous and no *MTL α*1, *α*2, **a**1, or **a**2 gene was detected in *L*. *elongisporus* isolates. For *C*. *orthopsilosis*, two isolates were *MTL***a** homozygous and five were *MTL*-heterozygous. Similarly, one *C*. *metapsilosis* isolate was *MTLα* homozygous whereas five were *MTL*-heterozygous. Isolate phenotypic switching analysis revealed potential phenotypic switching in the *MTLα* homozygous *C*. *metapsilosis* isolate, resulting in concomitant elongated cell formation. Minimum inhibitory concentrations of fluconazole (FLC) and FK506, alone or in combination, were determined by checkerboard assay, with data analyzed using the fractional inhibitory concentration index model. Synergistic or additive effects of these compounds were commonly observed in *C*. *parapsilosis* and *L*. *elongisporu*s isolates. No killer activity was observed in the studied isolates, as determined phenotypically. No significant difference in virulence was seen for the four species in a *Galleria mellonella* model (*P* > 0.05). In conclusion, our results demonstrated phenotypic switching of *C*. *metapsilosis* CBS 2315 and that FLC and FK506 represent a promising drug combination against *C*. *parapsilosis* and *L*. *elongisporu*s. The findings of the present study contribute to our understanding of the biology, diagnosis, and new possible treatments of the *C*. *parapsilosis* species group and *L*. *elongisporu*s.

## Introduction

The *Candida parapsilosis* group of species belonging to the human commensal mycoflora comprises three closely related taxa, *C*. *parapsilosis*, *C*. *orthopsilosis*, and *C*. *metapsilosis* [1]. *C*. *parapsilosis* is the most common pathogen within the group and is considered the most virulent. *Candida metapsilosis* is the least virulent species, with a low prevalence in human infections [2–4]. Thus, given the significant differences between species, it is not recommended to refer to a "species complex" for *C*. *parapsilosis* and its relatives [5]. Infections caused by *C*. *parapsilosis* and *C*. *orthopsilosis* are mainly described in severely ill patients from intensive care units, in low-birth-weight neonates, and in those receiving parenteral nutrition [6,7]. However, during the last decade, antifungal resistance to azoles and caspofungin has markedly increased worldwide among the entire group [8,9], which may contribute to increased prevalence.

*Lodderomyces elongisporus* was initially thought to represent the asexual state of *C*. *parapsilosis* [10]; however, subsequent small subunit rRNA gene sequencing revealed it to be a closely related but distinct species [11]. In a phylogenetic analysis, Riccombeni et al. [12] showed that *L*. *elongisporus* was classified within a clade of the *C*. *parapsilosis* species group and *C*. *albicans*, *C*. *dubliniensis*, and *C*. *tropicalis*, although in that clade, *L*. *elongisporus* is the only species that is able to produce ascospores [13,14]. Using large subunit rRNA gene sequencing, Lockhart et al. [15] described the first human *L*. *elongisporus* infections, which mostly occurred in patients from Mexico; notably, these isolates had initially been misidentified physiologically by the Vitek yeast identification system as *C*. *parapsilosis*. More recently, *L*. *elongisporus* has been shown to be globally distributed and human infections have been reported from the Middle East [16,17], Spain [18], and Japan [19]. However, compared to that in *C*. *parapsilosis*, antifungal resistance is fairly low in *L*. *elongisporus* [15–18].

Whereas mating has not yet been reported for the *C*. *parapsilosis* species group, *C*. *albicans* has been reported to have a parasexual cycle [20–23]. The mating-type like (*MTL*) locus of *C*. *albicans* is present as two idiomorphs: *MTL***a** and *MTLα* [24–26]. In contrast, only a single *MTL* idiomorph, *MTL***a**, has been identified in *C*. *parapsilosis* [27]. In comparison, *L*. *elongisporus* is reported to be in a homothallic sexual state, producing asci and ascospores in solo culture [13].

Switching between morphological phenotypes is common in pathogenic fungi and is hypothesized to be important in adapting to different environmental conditions [28]. *C*. *albicans* efficiently switches and is known to be present as yeast, hyphae, pseudohyphae, chlamydospores, and several yeast-like phases such as white, opaque, grey, and GUT phenotypes that have different virulence potentials and assist in the successful adaptation to different host niches [29]. The opaque phase also constitutes the mating-specialized form of *C*. *albicans* [30]. These are elongated and absorb phloxine B, producing pink colonies [31]. *C*. *parapsilosis* has also been reported to have different cell and colony morphologies, which exhibit different biofilm formation and agar invasion capabilities [32].

"Killer" characteristics were first observed in laboratory strains of *Saccharomyces cerevisiae* by Makower and Bevan [33], who defined the yeast phenotypes as killer, sensitive, and neutral. Killer yeasts secrete proteinaceous toxins that are lethal to sensitive strains, but to which the killer strains themselves are immune [34,35]. To date, killer yeasts have been reported in several genera, although the most widely studied killer systems are those of *S*. *cerevisiae* and *Kluyveromyces lactis*, with toxins that are RNA and DNA plasmid-encoded, respectively [36,37]. Growth inhibition evoked in sensitive strains by killer yeasts and their toxins has been proposed as a means of biotyping pathogenic *Candida* and *Cryptococcus* strains [38]. The killer system also enables species recognition within the *C*. *parapsilosis* species group, as only *C*. *metapsilosis* strains were shown to exhibit killer activity, in contrast to *C*. *parapsilosis* and *C*. *orthopsilosis* [39].

Published studies strongly suggest that *Galleria mellonella* provides a good alternative model for studying virulence in several fungi, including the major human fungal pathogens *Aspergillus* spp. [40], *Candida* spp. [41], and *Cryptococcus* spp. [42]. As the emerging resistance in the entire group of *C*. *parapsilosis* has led to difficult-to-treat infections [8,9], it is therefore important to understand the virulence potential of *C*. *parapsilosis* and its relatives. Furthermore, the development of drug combinations to address emerging antifungal resistance is critical for the management of patients, particularly in the case of invasive diseases [43–48]. Accordingly, Sun et al. [43] suggested the antifungal potential of a combination therapy with calcineurin pathway inhibitors (i.e., FK506, also known as tacrolimus) as a replacement for the activity of several azoles to combat azole resistance in *C*. *albicans*.

In the present study, we analyzed *C*. *parapsilosis* and its relatives to further characterize (i) the accuracy of their identification on BBL Chromagar *Candida* medium, (ii) their *MTL* genotypes and the occurrence of phenotypic switching, (iii) their killer activity and virulence in the *G*. *mellonella* model, and (iv) the ability of FK506 to enhance the isolate susceptibility to fluconazole (FLC).

## Materials and methods

### Isolates

The strains used in this study, i.e., *C*. *metapsilosis* (n = 6), *C*. *orthopsilosis* (n = 7), *C*. *parapsilosis* (n = 8), and *L*. *elongisporus* (n = 11), as well as their origin, identification number, and place of origin, are listed in S1 Table. *C*. *parapsilosis* species group isolates were provided by the culture collection of Centraalbureau voor Schimmelcultures (housed at Westerdijk Fungal Biodiversity Institute, Utrecht, the Netherlands) and *L*. *elongisporus* isolates were from the collection of the Molecular Genetics and Microbiology Department (Duke University, Durham, NC). All strains were subcultured on yeast extract-peptone dextrose agar (YEPD; Difco, Detroit, MI), at 37°C for 3 d, prior to analysis. The identities of all strains were verified prior to this study by sequencing the internal transcribed spacer region.

### Growth on chromogenic medium

All strains were inoculated in parallel onto BBL Chromagar Candida medium (Becton Dickinson and Company, Sparks, MD) and Sabouraud dextrose agar (SDA; Merck, Darmstadt, Germany), and incubated at 37°C for 72 h [15]. Following the incubation period, the isolates were evaluated on the basis of colony color. *C*. *albicans* SC5314 and *C*. *tropicalis* YJM57 were used as control strains.

### Genomic DNA isolation, polymerase chain reaction (PCR) amplification, and DNA sequencing

For genomic DNA isolation, all strains were collected directly from YEPD plates after 2 d of growth. Genomic DNA isolation was performed using the MasterPure Yeast DNA Purification Kit (Epicentre Biotechnologies, Madison, WI) according to the manufacturer’s instructions.

All PCR assays were conducted in a PTC-200 automated thermal cycler (BioRad, Hercules, CA); 300 ng DNA was used as a template for amplification in a 25 0μL reaction mixture containing 10 pM of each primer, 2 mM of each nucleotide (dATP, dCTP, dGTP, and dTTP), 2.5 μL 10 × Ex Taq buffer, 0.125 mL ExTaq polymerase (TaKaRa, Shiga, Japan), and an appropriate volume of distilled water. The primers and their sequences are specified in S2 Table.

The following conditions were used for standard PCR amplification: an initial 5 min denaturation at 94°C; followed by 36 cycles of denaturation for 1 min at 94°C, annealing for 1 min at 57°C, and an extension for 1 min at 72°C. The amplification was completed with a final extension period of 10 min at 72°C. For amplification using degenerate primers, a touchdown protocol was applied (5 min at 94°C; 24 cycles of 45 s at 94°C, 45 s at 66°C–54°C step-down at 0.5°C every cycle, 1 min at 72°C; 16 cycles of 45 s at 94°C, 45 s at 54°C, 1 min at 72°C, and a final extension step of 10 min at 72°C). Sterile water instead of DNA served as a negative control in each assay. PCR products were analyzed on 1% agarose gels.

Amplicons to be sequenced were purified using the QIAquick PCR Purification Kit (Qiagen, Germantown, MD) as recommended by the manufacturer. Both strands of PCR products were sequenced using BigDye Terminator version 3.1 cycle sequencing ready reaction mix (Applied Biosystems, Foster City, CA). Sequencing products were resolved using an ABI 3130 automated sequencer (Applied Biosystems) and the sequences were assembled using Sequencher 4.8. software (Gene Code Corporation, Ann Arbor, MI).

### Switching test

To examine the occurrence of phenotypic switching, the isolates were grown on plates of supplemented Lee’s agar medium for 5 d and then plated on synthetic complete (SC) medium containing 5 μg/mL phloxine B; they were then further grown for 7 d at 26°C and 30°C [30]. *C*. *albicans* WO-1 white and *C*. *albicans* WO-1 white opaque strains obtained from the Duke University Molecular Genetics and Microbiology Department collection were used as control strains.

### Killer activity/sensitivity assay

*C*. *parapsilosis* species group and *L*. *elongisporus* isolates were assayed to determine their killer/sensitivity phenotypes. The killer sensitivity of *C*. *parapsilosis* species group and *L*. *elongisporus* strains was assayed by mixing each isolate with YEPD-MB agar [0.3% yeast extract, 0.3% malt extract, 0.5% peptone, 2% glucose, 2% agar, and 0.003% methylene blue (MB); adjusted to pH 4.5 with 0.1 M citrate-phosphate buffer] to a final concentration of 10^6^ cells/mL and by streaking the known killer and sensitive isolates on the surface of plates. The plates were incubated at 26°C for up to 72 h. The sensitivity test was considered positive if killer strains showed a clear inhibition zone surrounded by a blue halo [39]. RNA extraction was performed using TRIzol reagent (Thermo Fisher Scientific, Waltham, MA) according to a protocol provided by the manufacturer. *S*. *cerevisiae* strains were used as controls. Products were analyzed on 1% agarose gels.

### Evaluation of virulence using the *G*. *mellonella* model

Isolates were pre-grown on YPD agar for 24 h at 37°C and then harvested by gentle scraping of the colony surfaces with plastic loops and washed three times in sterile phosphate-buffered-saline (PBS). Cell suspensions were counted using a hemocytometer, and cell density adjusted to 10^6^ cells/μL with sterile PBS. The virulence of each isolate was tested in 15 *G*. *mellonella* larvae. Cell suspensions in sterile PBS (4 μL) were injected via the last left rear proleg, using a 100 μL Hamilton syringe with dispenser. The syringe was rinsed several times with 70% ethanol, followed by a PBS rinse, prior to injecting each larva. The control group of larvae was inoculated with sterile PBS. Inoculated larvae were incubated at 37°C and the number of dead animals was monitored daily [4].

### Determination of antimicrobial drug resistance

FLC (Sigma, St. Louis, MO) and FK506 (Sigma) were diluted in sterile water according to the Clinical and Laboratory Standards Institute (CLSI) protocol [49]. Serial two-fold dilutions of each drug were prepared in RPMI 1640. Synergy testing of FLC and FK506 against *C*. *parapsilosis* species group and *L*. *elongisporus* strains was assessed by the checkerboard method [50]. The test was performed as a microdilution assay, in duplicate for each fungal strain. The minimum inhibitory concentrations (MIC) were interpreted according to the CLSI guidelines [49]. To evaluate the effect of the combinations of FLC and FK506, the fractional inhibitory concentration (FIC) was calculated for each antifungal agent in every combination. The following formulas were used to calculate the FIC index: FIC of drug A, MIC (drug A in combination)/MIC (drug A alone); FIC of drug B, MIC (drug B in combination)/MIC (drug B alone); FIC index, sum of FIC of drug A and FIC of drug B. Antifungal combinations were evaluated based on FIC index ranges, as follows: synergistic, if ≤ 0.5; additive, if > 0.5 but < 1; no effect, if ≥ 1 but < 4; and antagonistic if ≥ 4 [51,52].

### Statistical analysis

The Kaplan-Meier test was performed to assess the statistical significance of differences in survival among groups. Survival curves were analyzed using Minitab v. 16.1 software with the log-rank (Mantel-Cox) test; *P* < 0.05 was considered statistically significant.

## Results and discussions

In this study, the identifying characteristics, *MTL* genotypes, phenotypic switching, and susceptibility profiles of *C*. *parapsilosis* species group and *L*. *elongisporus* reference isolates were analyzed to gain insight into their pathobiology and virulence mechanisms.

### Chromogenic medium

**Fig 1:**
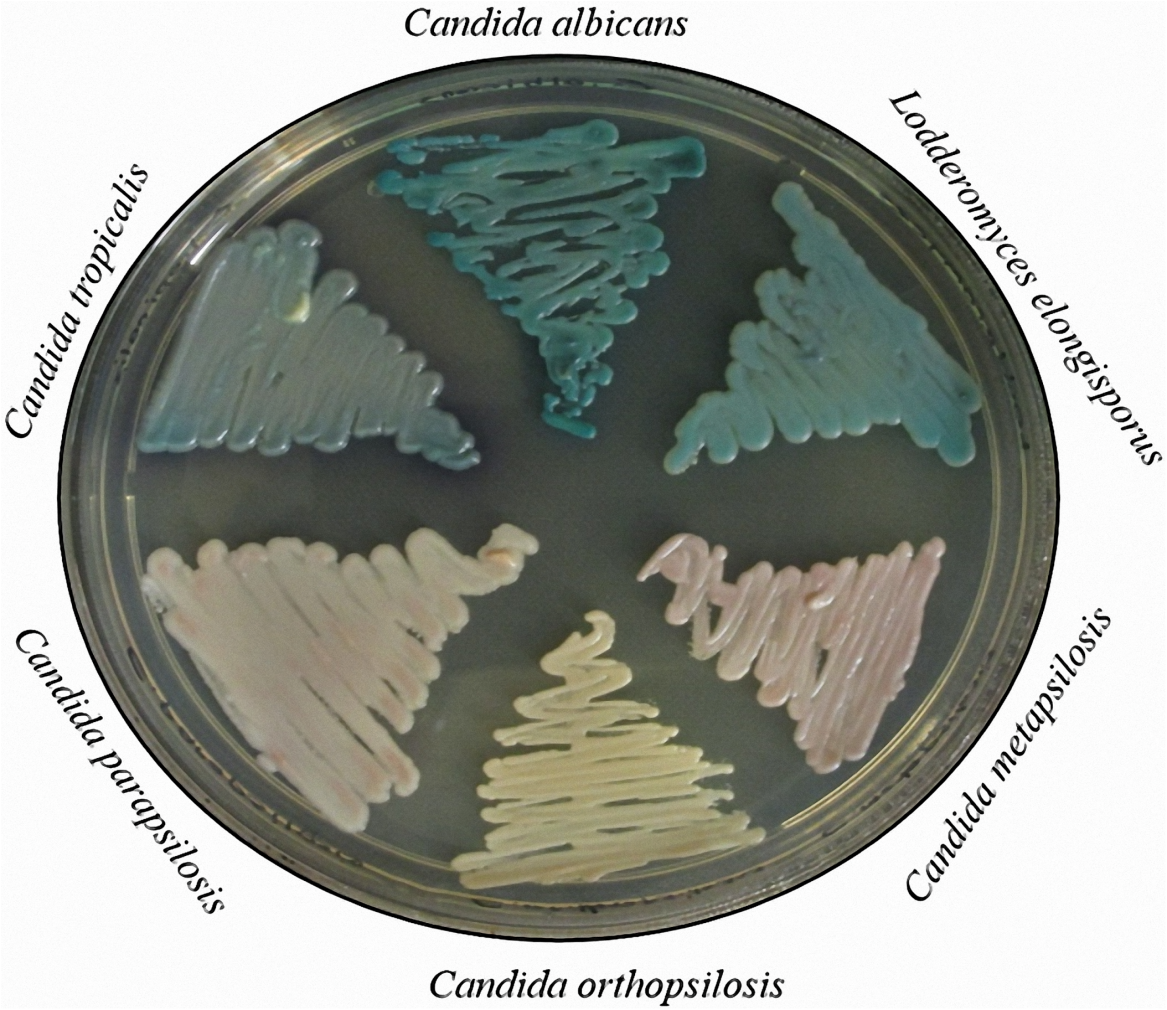
*Candida albicans* SC 5314, *Candida tropicalis* YJM 57, *Lodderomyces elongisporus* 7660, *Candida metapsilosis* CBS 2315, *Candida orthopsilosis* CBS 107.41, and *Candida parapsilosis* CBS 2315 were grown on BBL Chromagar Candida medium at 37°C for 3 d and photographed.

The adequacy of BBL Chromagar Candida medium for the initial identification of reference strains was evaluated. All *C*. *parapsilosis* and *C*. *metapsilosis* isolates formed light pink colonies; *C*. *orthopsilosis* formed ivory colonies; and *L*. *elongisporus* formed blue/green colonies similar to *C*. *tropicalis* and *C*. *albicans* on this medium. Representative colonies on the chromogenic medium are shown in Fig 1.

The utility of chromogenic media for the screening and initial identification of yeasts in polyfungal clinical materials is well established [53–56]. *C*. *parapsilosis* characteristically produces pink or lavender colonies on BBL CHROMagar Candida medium, whereas *L*. *elongisporus* isolates were reported to form colonies with a distinct turquoise color [15]. However, *C*. *parapsilosis* and *C*. *metapsilosis* isolates formed light pink colonies, rendering their presumptive identification problematic.

### Determination of MTL genotypes

Sexual reproduction is important in the evolution of fungal pathogens. In particular, pathogenic fungi are hypothesized to restrict sexual reproduction so as not to disrupt well-adapted pathogenic genotypes; however, they maintain the potential for sexual reproduction to cope with stressful conditions such as antimicrobial therapy [57]. The *MTL* locus is responsible for the determination of cell identity and regulation of mating in *C*. *albicans* and related species [25]. In *C*. *albicans*, there are two versions of *MTL*: *MTL***a** which harbors the transcription factor genes **a**1 and **a**2; and *MTLα*, which encodes the *α*1 and *α*2 regulatory elements [24–26]. Both idiomorphs also possess **a** or *α* versions of additional genes that have no known function in mating, such as *PAB*, *OBP*, and *PIK* [20]. In *C*. *albicans*, *α*1 and **a**2 activate the *α*- and **a**-specific genes, respectively; the **a**1/*α*2 heterodimer plays a role both in the regulation of mating and in white/opaque switching, by repressing mating and limiting switching to the opaque phase in **a**/*α* cells by inhibiting White-opaque regulator 1 (*WOR1*), which is regulated by other factors as well [29,58,59]. In comparison, *C*. *orthopsilosis* has an *MTL* locus very similar to that of *C*. *albicans* and harbors both *MTL***a** and *MTLα* idiomorphs, whereas only the *MTL***a** idiomorph has been identified to date in *C*. *parapsilosis* [27].

**Fig 2:**
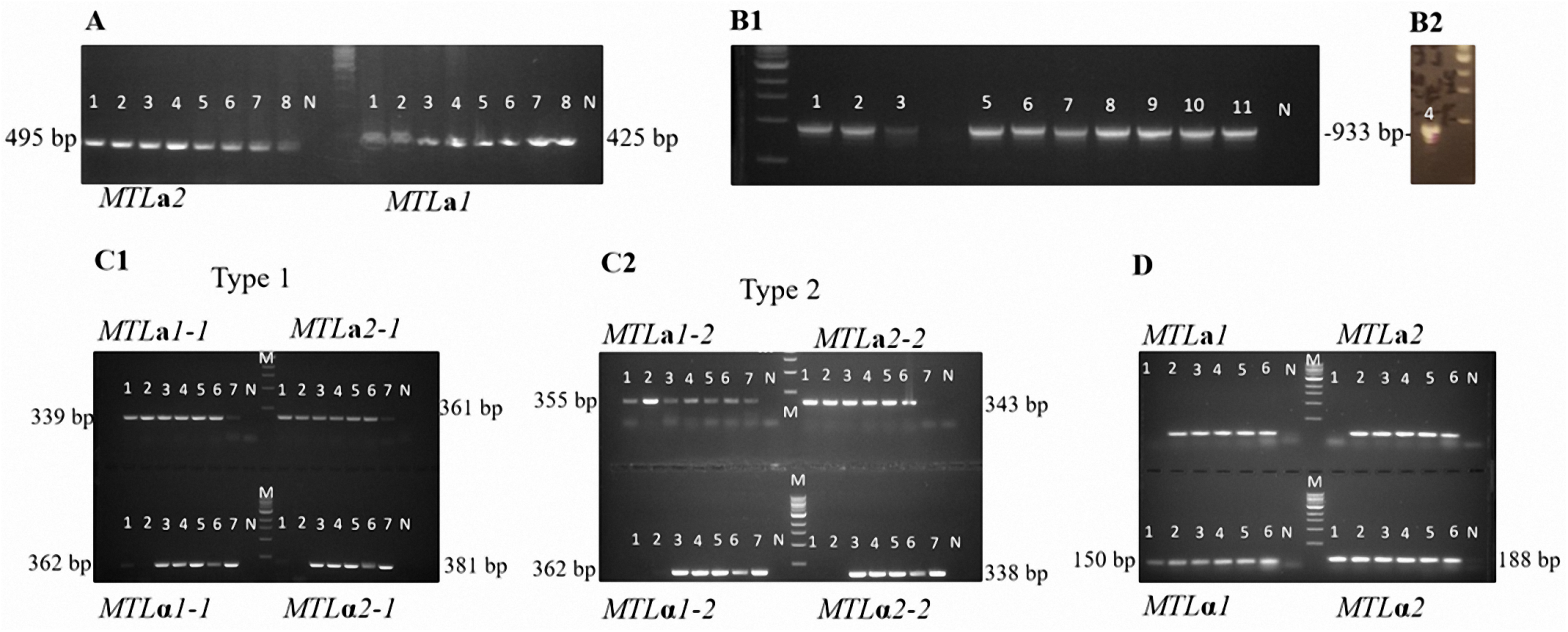
Determination of the *MTL* genotypes by PCR, **(a)** *Candida parapsilosis* isolates (1–8 referring to CBS 8836, CBS 7248, CBS 2915, CBS 604, CBS 2216, CBS 8181, CBS 125.41, and CBS 1954), the 425 bp *MTL***a**1 and the 495 bp *MTL***a**2 products were obtained for all isolates; **(b1** and **b2)** *Lodderomyces elongisporus* isolates (1–11 referring to the isolates 7660, 7661, 7663, 7665, 7666, 7668, 7669, 7670, 7672, 7673, and 7675), the 933 bp PCR product obtained for all isolates indicates the absence of any *MTL* transcription factor gene in between *PIK***a** and orf19.3202; **(c)** *Candida orthopsilosis* isolates (1–7 referring to CBS 107.41, CBS 107.42, CBS 109.06, CBS 8825, CBS 107.43, CBS 9894, and CBS 2212) were screened with primers designed based on *C*. *orthopsilosis* type 1 **(c1)** and type 2 *MTL* sequences **(c2)**; both primer pairs worked for the isolates although band brightness’ varied owing to differences among type 1 and type 2 sequences. With CBS 107.41 and CBS 107.42, only *MTL***a**1 and *MTL***a**2 PCR products were obtained indicating that these isolates are *MTL***a** homozygotes; the other isolates were found to be heterozygous for *MTL*; **(d)** *Candida metapsilosis* isolates (1–6 referring to CBS 2315, CBS 107.47, CBS 109.07, CBS 111.27, CBS 1046, and CBS 2916), whereas only *MTLα*1 (150 bp) and *MTLα*2 (188 bp) PCR products were obtained with CBS 2315 indicating *MTLα* homozygosity; all *MTL* gene products were obtained for the other isolates showing that they are *MTL* heterozygous. M, Marker; NC, Negative control.

Mating type genes of the isolates were determined by PCR. As no *MTL α*1, *α*2, **a**1, or **a**2 genes have been detected in *L*. *elongisporus* to date and no *C*. *parapsilosis MTLα* idiomorph is known, degenerate primers to amplify the *MTL* transcription factor genes **a**1, **a**2, *α*1, and *α*2 were designed to search for the presence of these genes. In addition, specific primers were designed to amplify the **a**2 gene and **a**1 pseudogene in *C*. *parapsilosis*. PCR screening of eight *C*. *parapsilosis* isolates revealed only the presence of **a**1 and **a**2 genes (Fig 2A), indicating that these isolates all bear the *MTL***a** idiomorph. An *MTLα* idiomorph was not detected using the degenerate primers.

*L*. *elongisporus* is the only close relative of *C*. *parapsilosis* reported to exhibit a homothallic sexual state [13]. However, in the sequenced strain and in seven additional isolates of *L*. *elongisporus*, a genomic region that is syntenic to the *MTL* locus of other *Candida* species only has the *MTL***a** versions of *PAB*, *OBP*, and *PIK* genes, and does not contain any transcription factor genes [25]. Considering the genome analysis and molecular data, it was suggested that *L*. *elongisporus* might not have a sexual cycle or the sexual cycle might function in a manner independent of *MTL* [25].

In this study, for *L*. *elongisporus*, primers were designed to bind within *PIK***a** and orf19.3202 (encoding a hypothetical protein ortholog of *C*. *albicans* CAALFM_C501730WA, located just outside the *MTL* locus in *C*. *albicans* and other closely related species), respectively [25], to check for the presence of mating genes between these sites. In the absence of any such genes, a 933 bp PCR product was predicted, as in the sequenced isolate [25]. All *L*. *elongisporus* isolates yielded 933 bp amplicons, indicating the absence of an *MTL* idiomorph (Fig 2B1 and 2B2). Furthermore, neither an **a** nor *α* gene was detected in the isolates using degenerate primers.

In *C*. *orthopsilosis*, *α*1, *α*2, **a**1, and **a**2 genes were screened using specific primers. Only **a**1- and **a**2-specific PCR product bands were obtained from isolates CBS 107.41 and CBS 107.42; all the expected gene PCR product bands were obtained from the remaining five isolates. This indicated **a**/**a** homozygosity of the former two isolates and **a**/*α* heterozygosity of the remainder (Fig 2C1 and 2C2). In comparison, in a study by Sai et al. [27], only two of 16 *C*. *orthopsilosis* isolates were found to be *MTL* heterozygous, whereas nine were *MTL***a** homozygous, and five were *MTLα* homozygous.

According to Pryszcz et al. [60], the *MTLα* locus of *C*. *metapsilosis* is very similar to that of *C*. *albicans* in structure, encoding the genes *MTLα*1, *MTLα*2, *OBPα*, *PIKα*, and *PAPα*. The *MTL***a** locus, however, harbors *MTLα*2, *OBPα*, and *PIKα* in addition to the **a**-specific genes *MTL***a**1 and *MTL***a**2. For *C*. *metapsilosis* isolates, primers designed by Pryszcz et al. [60] were used to amplify the *α*1, *α*2, **a**1, and **a**2 genes. Whereas PCR products for each gene were obtained from five *C*. *metapsilosis* isolates, only the *α*1 and *α*2 products were obtained from CBS 2315. Thus, CBS 2315 carried the *α*/*α* genotype and the other five isolates were **a**/*α* (Fig 2D). Similarly, 10 out of 11 *C*. *metapsilosis* isolates analyzed by Pryszcz et al. [60] were *MTL* heterozygous.

### Phenotypic switching within the isolate set

**Fig 3:**
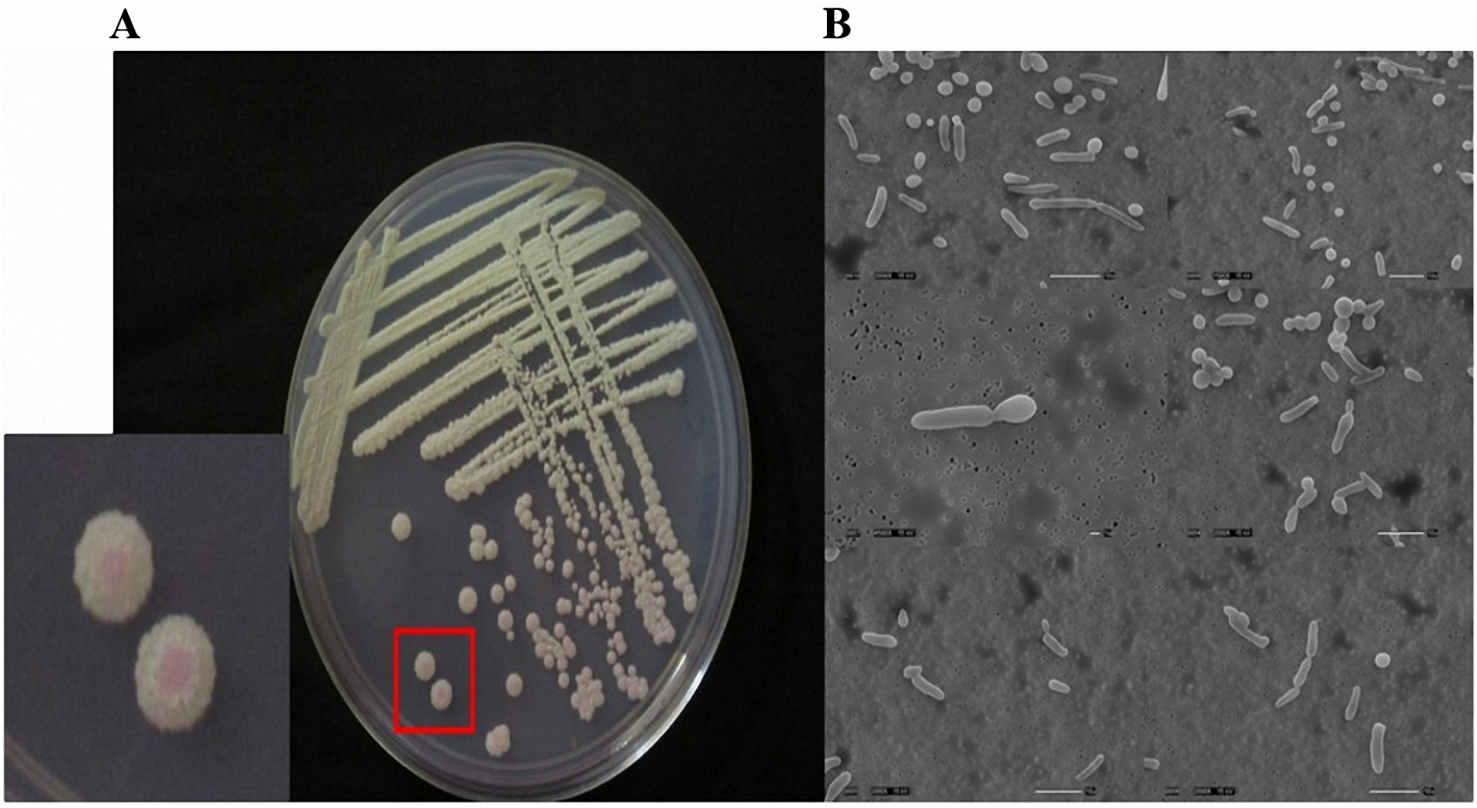
Phenotypic switching test with *Candida metapsilosis* CBS 2315 (MAT *α*/*α*). **(a)** Pink colonies indicating phenotypic switching (red box), grown at 30°C for 7 d on SC medium containing 5 μg/mL phloxine B; **(b)** elongated cells analyzed by scanning electron microscopy (Scale bar, 10 μm).

Previous studies have identified different colony and cell morphologies of *Candida* spp., especially *C*. *albicans* and *C*. *parapsilosis* isolates. Although pseudohyphae formation has been observed in *C*. *parapsilosis* and *C*. *albicans* as well as in *C*. *orthopsilosis*, it was not observed in *C*. *metapsilosis* [4,61,62]. In contrast, in the present study we detected phenotypic switching in *C*. *metapsilosis* CBS 2315, the isolate carrying the *MTLα*/*MTLα* genotype, at 37°C. The switching led to the formation of pink colonies in the presence of phyloxine B, which contained elongated cells revealed by scanning electron microscopy (Fig 3).

### Killer activity of the isolates

A total of 32 isolates were analyzed by the killer/sensitivity phenotype test. Briefly, 22 out of 32 isolates produced weak blue halos around their colonies; nine isolates did not produce such halos. *C*. *parapsilosis* CBS 2915 produced a weak blue halo around both the killer and sensitive strains. However, no RNA band was observed on a 1% agarose gel, indicating the absence of killer activity due to a dsRNA virus.

*C*. *parapsilosis* has been reported to be a killer yeast [63], although killer strains were reported to represent less than 3% of clinical isolates of the species [64]. Killer activity was found to be expressed at 25°C, whereas isolates of *C*. *parapsilosis* and *C*. *orthopsilosis* did not show this activity at 25°C [62]. Because the killer toxin is thermolabile [34], wild-type killers exhibit very little killing activity at 30°C and are normally tested at 20°C. Therefore, in the present study, the isolates were analyzed at 26°C, 30°C, and 37°C; however, no killer activity was detected.

### Virulence of the isolates in the *G*. *mellonella* model

**Fig 4:**
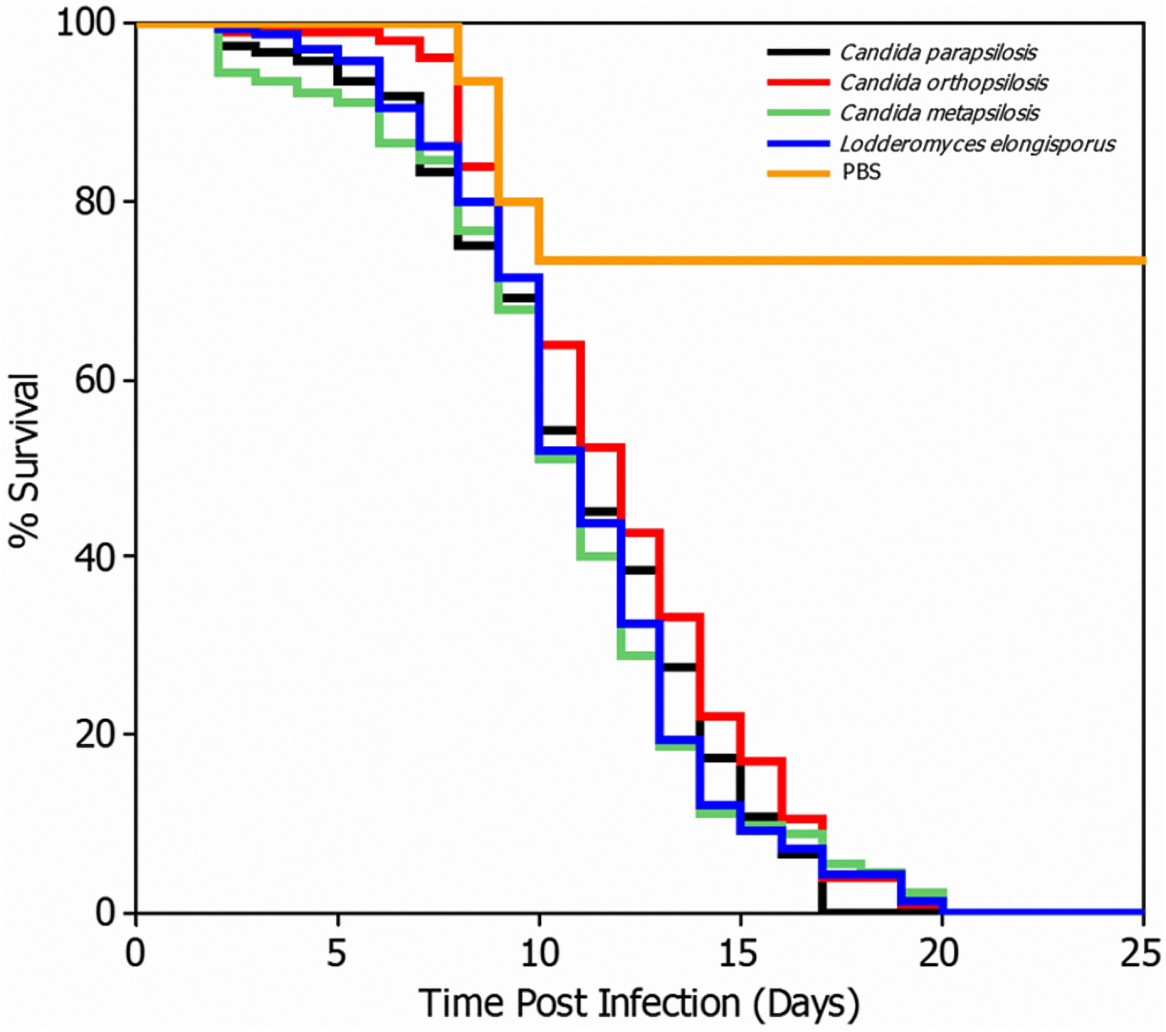
*Galleria mellonella* survival curves after larval infection with the indicated *Candida parapsilosis* (blue line), *Candida orthopsilosis* (green line), *Candida metapsilosis* (purple line), and *Lodderomyces elongisporus* (turquoise line) species, or with PBS as a control (red line). A total of 1 × 10^6^ cells were used to infect the larvae and animal survival was observed at 37°C. All study isolates were tested.

Virulence of the 32 study isolates was compared using the *G*. *mellonella* model (S3 Table). We observed no significant differences among the *C*. *parapsilosis* species group and their closely related species *L*. *elongisporus* (*P* > 0.05); however, significant differences were detected with the PBS control group (*P <* 0.05; Fig 4).

*C*. *orthopsilosis* was reported by Gago et al. [4] to represent the most virulent species of the *C*. *parapsilosis* species group in the *G*. *mellonella* model, followed by *C*. *parapsilosis* and *C*. *metapsilosis*, with a median survival time of 2.3, 2.6, and 4.5 d, respectively. It has been previously suggested that these scores could not be related to the growth rate of *Candida* spp. [65]. Notably, however, *C*. *metapsilosis* strains were more effectively phagocytosed by *G*. *mellonella* hemocytes than in *C*. *parapsilosis* and *C*. *orthopsilosis* (*P* < 0.05) [4]. Furthermore, hyphae or pseudohyphae formation was less frequent in *C*. *metapsilosis* isolates than in *C*. *parapsilosis* and *C*. *orthopsilosis* (*P* < 0.05) [4]. In another study, one oral and one systemic isolate of *C*. *parapsilosis*, both susceptible to FLC and amphotericin B, killed the *G*. *mellonella* larvae within 18 h and 21 h, respectively, which suggested that the clinical origin of the strain is not important for virulence (*P* = 0.6) [4]. However, in contrast to these previous reports [4,65], which did not differ with respect to inoculum size in comparison with the present study (1 × 10^6^ cells/larvae), we did not observe a difference in virulence using this model among the studied fungi.

### Antimicrobial resistance of the isolates

MIC values of FLC and FK506 were determined in synergy tests using the checkerboard assay (Table 1). FIC indices were calculated by considering all combinations of drugs where no visible growth was observed. The results were as follows: for *C*. *metapsilosis*, 33.3% synergy and 66.7% indifferent effect; for *C*. *orthopsilosis*, 14.3% synergy, 14.3% additive effect, and 71.4% indifferent effect; for *C*. *parapsilosis*, 25% synergy, 62.5% additive effect, and 12.5% indifferent; and for *L*. *elongisporus*, 36.4% synergy, 45.4% additive effect, and 18.2% indifferent effect.

**Table 1:** Synergy testing of fluconazole (FLU) and FK506 against *Candida parapsilosis* species group and *Lodderomyces elongisporus* strains.

Taxon Name	Reference no	Minimum inhibitory concentrations (MIC; μg/ml)				Fractional inhibitory Outcome concentration (FIC) Index	
				Best Combined			
		FLU	FK506	FLU	FK506		
*C*.*metapsilosis*	CBS 2315	1	>16	1	0.03125	1.0019	Indifferent
	CBS 107.47	1	>16	1	0.125	1.007	Indifferent
	CBS 109.07*	4	>16	1	2	0.375	Synergy
	CBS 111.27	16	>16	8	8	1	Indifferent
	CBS 107.46	8	>16	2	0.125	0.2578	Synergy
	CBS 2916	16	>16	16	8	1.5	Indifferent
*C*.*orthopsilosis*	CBS 107.41	1	>16	1	0.03125	1.0019	Indifferent
	CBS 107.42	4	>16	1	0.125	0.2578	Synergy
	CBS 109.06*	2	>16	2	0.25	1.015	Indifferent
	CBS 8825	1	>16	2	0.125	2.0078	Indifferent
	CBS 107.43	16	>16	16	0.125	1.0078	Indifferent
	CBS 9894	4	>16	4	0.0625	1.0039	Indifferent
	CBS 2212	8	>16	4	0.125	0.5078	Additive
*C*.*parapsilosis*	CBS 8836	1	>16	0.5	0.03125	0.5019	Additive
	CBS 7248	0.5	>16	0.25	0.125	0.507	Additive
	CBS 2915	1	>16	2	0.15625	2.0098	Indifferent
	CBS 604	4	>16	2	0.03125	0.5019	Additive
	CBS 2216	1	>16	0.25	1	0.3125	Synergy
	CBS 8181	4	>16	1	0.03125	0.2519	Synergy
	CBS 125.41	2	>16	1	0.03125	0.5019	Additive
	CBS 1954*	4	>16	2	0.03125	0.5019	Additive
*L*.*elongisporus*	7660	1	>16	0.5	0.03125	0.5019	Additive
	7661	1	>16	0.5	0.03125	0.5019	Additive
	7663	1	>16	0.25	0.0625	0.2539	Synergy
	7665	1	>16	1	0.0625	1.0039	Indifferent
	7666	1	>16	0.5	4	0.75	Additive
	7668	1	>16	0.5	0.03125	0.50195	Additive
	7669	1	>16	1	0.015625	1.00098	Indifferent
	7670	2	>16	0.5	0.0625	0.2539	Synergy
	7672	2	>16	0.5	0.03125	0.2519	Synergy
	7673	2	>16	0.25	4	0.375	Synergy
	7675	1	>16	0.5	1	0.5625	Additive

CBS, Centraalbureau voor Schimmelcultures.

*, Type strain.

The intrinsic antifungal resistance of *Candida* spp. constitutes a major issue related to the therapeutic management of infections and has required the utilization of combinatorial therapy, such as with FK506. For example, Chen et al. [44] reported that posaconazole exhibits an *in vitro* and *in vivo* synergistic antifungal activity with caspofungin or FK506 against *C*. *albicans* isolates. Cruz et al. [45] also observed that FK506 was synergistic with FLC against azole-resistant *C*. *albicans* mutants, against other *Candida* species, or when combined with different azoles. Notably, Li et al. [46] observed that a combination of FLC and FK506 might represent a promising approach toward overcoming the intrinsic resistance of *Candida krusei* to FLC. Denardi et al. [47] also investigated the *in vitro* interaction of FK506 and four azole compounds against 30 clinical FLC-susceptible or FLC-resistant *Candida glabrata* isolates using the microdilution checkerboard method. In particular, they detected a promising synergistic effect against FLC-resistant *C*. *glabrata* isolates of FK506 combined with ketoconazole (77%), itraconazole (73%), voriconazole (63%), and FLC (60%). In contrast, FK506 showed no activity against 30 clinical FLC-susceptible and FLC-resistant *Trichosporon asahii* isolates, with MICs ≥ 64 μg/ml. However, a pronounced synergistic interaction of FK506 in combination with amphotericin B (96.7%) and caspofungin (73.3%) was observed, although low rates of synergism were observed with FLU (40%) and itraconazole (10%) [48].

In the present study, we observed synergistic and additive, or indifferent, effects of FLC and FK506 against *C*. *parapsilosis* and related species including *L*. *elongisporus*; however, antagonistic activity was not observed. Previously, nine human *L*. *elongisporu*s isolates were tested against FLC, amphotericin B, caspofungin, anidulafungin, and micafungin, all of which exhibited low MICs, as determined by the CLSI microdilution method [15]. Consistent with these findings [15], we observed low FLC MICs for *L*. *elongisporu*s, for which no established breakpoint values are currently available. The present study also demonstrated that synergistic and additive effects of FLC and FK506 were more apparent against *C*. *parapsilosis* (87.5%) and *L*. *elongisporu*s (81.8%) than other isolates, and that the combination was likely to have no effect against *C*. *orthopsilosis* (71.4%) and *C*. *metapsilosis* (66.7%). However, a limitation of the present study is that only a small number of isolates was tested. Hence, no universal conclusion may be reached regarding the data obtained herein.

In conclusion, we determined the *MTL* genotypes of a set of reference isolates of the *C*. *parapsilosis* species group and detected an *MTLα* homozygous *C*. *metapsilosis* isolate that underwent phenotypic switching and produced elongated cells. Furthermore, we observed no significant difference in virulence among the four species, using a *G*. *mellonella* model. We suggest that the FLC/FK506 combination may be promising as a therapeutic strategy against *L*. *elongisporu*s and *C*. *parapsilosis* isolates, but not against *C*. *orthopsilosis* and *C*. *metapsilosis*. *In vitro* assessment in an experimental model is required to verify the efficacy of this drug combination. Overall, these new data may be used to guide strategies for combating these pathogens in the clinic.

## Supporting information

S1 Table Isolates used in this study. (DOC)

S2 Table Primers used in this study. (DOCX)

S3 Table The survival data for *Galleria* larvae infected with different fungal species. (XLSX)
