# Supplementary material for: MTL genotypes, phenotypic switching, and susceptibility profiles of Candida parapsilosis species group compared to Lodderomyces elongisporus
Source: PLoS One. 2017 Aug 3;12(8):e0182653. doi: 10.1371/journal.pone.0182653 (PMC5542550; doi:10.1371/journal.pone.0182653)
Supplement: S1 Table — (DOC) [file pone.0182653.s001.doc]

**S1 Table.** Isolates used in this study.

| **Taxon Name** | **Reference no** | **Sample Type** | **Sample Origin** | **Sample Point** | **Country** |
| --- | --- | --- | --- | --- | --- |
| *C.metapsilosis* | CBS 2315 | Clinical | Human | Sputum | Italy |
|  | CBS 107.47 | Clinical | Human | Mouth of a Titetan male nail | Belgium |
|  | CBS 109.07* | Clinical | Human | Unknown | USA |
|  | CBS 111.27 | Unknown | Unknown | Unknown | Unknown |
|  | CBS 107.46 | Unknown | Unknown | Unknown | Unknown |
|  | CBS 2916 | Unknown | Unknown | Unknown | Norway |
| *C.orthopsilosis* | CBS 107.41 | Clinical | Human | Moss Nail | Belgium |
|  | CBS 107.42 | Clinical | Human | Nail | Belgium |
|  | CBS 109.06* | Clinical | Human | Vaginal | USA |
|  | CBS 8825 | Environmental | Plant | Unknown | Australia |
|  | CBS 107.43 | Environmental | Scarabaeidae ex leaf | Vaginal | USA |
|  | CBS 9894 | Environmental | Insect | Unknown | Panama |
|  | CBS 2212 | Environmental | Teabee | Unknown | Indonesia |
| *C.parapsilosis* | CBS 8836 | Clinical | Human | Blood | USA |
|  | CBS 7248 | Clinical | Animal | Udder of cow with subclinical mastitis | New Zealand |
|  | CBS 2915 | Clinical | Human | Unknown | Norway |
|  | CBS 604 | Clinical | Human | Unknown | Puerto Rico |
|  | CBS 2216 | Environmental | Food | Pickling vat with 10% brine | USA |
|  | CBS 8181 | Environmental | Plant | Rotten trunk of *Nothofagus dombeyi* | Chile |
|  | CBS 125.41 | Environmental | Fruit | Traditionally fermented *Ziziphus mauritiana* fruit | Zimbabwe |
|  | CBS 1954* | Environmental | Fruit | Unknown | Italy |
| *L.elongisporus* | 7660 | Clinical | - | - | Mexico |
|  | 7661 | Clinical | - | - | Malaysia |
|  | 7663 | Clinical | - | - | USA |
|  | 7665 | Clinical | - | - | Venezuela |
|  | 7666 | Clinical | - | - | Venezuela |
|  | 7668 | Clinical | - | - | Italy |
|  | 7669 | Clinical | - | - | Mexico |
|  | 7670 | Clinical | - | - | Mexico |
|  | 7672 | Clinical | - | - | Mexico |
|  | 7673 | Clinical | - | - | Mexico |
|  | 7675 | Clinical | - | - | Mexico |

CBS, Centraalbureau voor Schimmelcultures; *, Type strain.
