# Supplementary material for: MTL genotypes, phenotypic switching, and susceptibility profiles of Candida parapsilosis species group compared to Lodderomyces elongisporus
Source: PLoS One. 2017 Aug 3;12(8):e0182653. doi: 10.1371/journal.pone.0182653 (PMC5542550; doi:10.1371/journal.pone.0182653)
Supplement: S2 Table — (DOCX) [file pone.0182653.s002.docx]

**S2 Table.** **Primer used in this study.**

|  | **Primer name** | **Primer sequence** | **Expected**  **product size** |
| --- | --- | --- | --- |
|  | ITS1 | 5'- TCCGTAGGTGAACCTGCGG-3' | ~450 bp |
|  | ITS4 | 5'-TCCTCCGCTTATTGATATGC-3' |  |
| ***L. elongisporus*** | Le-PIKa-F | 5'-TTGATGCTTTTGCAGAGACG-3' | 933 bp |
|  | Le-Orf19.3202-R | 5'-TACCACGAAACACCCACTGA-3' |  |
| ***C. metapsilosis*** | MTLα1_1F* | 5'-GCCGCCTGAGAAGTATGAAG-3' | 150 bp |
|  | MTLα1_1R* | 5'-TTGGTGACCACAGGAAAACA-3' |  |
|  | MTLα2_1F* | 5'-GTGCTCCTCAAGCACAATCA-3' | 188 bp |
|  | MTLα2_1R* | 5'-GTCGCCCAGACAACTCTAGC-3' |  |
|  | MTL**a**1_1F* | 5'-GCTTGAGTGGGGATTGAGTC-3' |  |
|  | MTL**a**1_1R* | 5'-AATCGTTTTCGGGGTTTTCT-3' |  |
|  | MTL**a**2_1F* | 5'-TCTCCGGATTCGTTCAATTC-3' |  |
|  | MTL**a**2_1R* | 5'-CTTGACCCCAAAGCTTTCAA-3' |  |
| ***C. parapsilosis*** | Cp-**a**2-F | 5'-TCCCCATCCTGTCAATCAAT-3' | 495 bp |
|  | Cp-**a**2-R | 5'- TCGAATTTTGGGTCTCTTCG-3' |  |
|  | Cp-**a**1pseudo-F | 5'- TAAACCCAAGGTTCGAATGG-3' | 425 bp |
|  | Cp-**a**1pseudo-R | 5'- TTCGCGAACCATAATGAGAG-3' |  |
| ***C. orthopsilosis*** | Co-type1-α1-F | 5'-GATTCCGAAGGATTTCGTCA-3' | 362 bp |
|  | Co-type1-α1-R | 5'-ACTGCTCCCAGACATCCCTA-3' |  |
|  | Co-type1-α2-F | 5'-GACGCTTGCTTGGCTTAGAC-3' | 381 bp |
|  | Co-type1-α2-R | 5'-CCGTAGGTCAAGCATCAACA-3' |  |
|  | Co-type1-**a**1-F | 5'-GAGGGCAAGGCTTTGTTAGA-3' | 339 bp |
|  | Co-type1-**a**1-R | 5'-CTGAAATTGAAACCCGTCGT-3' |  |
|  | Co-type1-**a**2-F | 5'-GGCCGAGAAGGGAAATAAGT-3' | 361 bp |
|  | Co-type1-**a**2-R | 5'-CCAGGCATGTTTTCGTCTTT-3' |  |
|  | Co-type2-α1-F | same as type 1 | 362 bp |
|  | Co-type2-α1-R | 5'-ACTGCTCCCAGATGTCCCTA-3' |  |
|  | Co-type2-α2-F | 5'-AGACGCTTGCTTGGCTTAGA-3' | 338 bp |
|  | Co-type2-α2-R | 5'-GTGAACTTGTGTTCGCCAAA-3' |  |
|  | Co-type2-**a**1-F | 5'-TCAACAGCGCTCTTTTGCTA-3' | 355 bp |
|  | Co-type2-**a**1-R | 5'-CAATCAACCCCCTCTCTTCA-3' |  |
|  | Co-type2-**a**2-F | 5'-CTCCACAGCATCGACTGAGA-3' | 343 bp |
|  | Co-type2-**a**2-R | 5'-TTCTGCCGACTTGCAAATAA-3' |  |
| **Degenerate** | Alpha1-F-deg | 5'-C(T/A)ATIAA(T/C)TCITT(T/C)AT(C/T)GCITT-3' | ~120 bp |
| **primers** | Alpha1-R-deg | 5'-TT(G/A)TAI(A/G)AT(A/T)(A/G)IG(T/C)GTA(C/T)TGIT(T/C)CCA-3' |  |
|  | Alpha2-F-deg | 5'-TCIAA(A/G)TCICA(A/G)(A/G)T(A/C)CAAAATTGGTA-3' | ~100 bp |
|  | Alpha2-R-deg | 5'-T(C/T)TC(T/C)TTTCTTCTTC(G/T)ATTIGA(A/T)(A/G)(C/T)CCT-3' |  |
|  | **a**1-F-deg | 5'-C(C/T)ICIAA(G/A)GA(G/A)(C/T)TTCTTG(A/T)(A/G)A-3' | ~110 bp |
|  | **a**1-R-deg | 5'-AA(G/T)ACATAC(C/A)CAIAC(C/T)C(T/G)(A/T)ATTT-3' |  |
|  | **a**2-F-deg | 5'-AG(G/A)AA(T/C)AG(T/C)TT(C/T)AT(C/T)ATTGC(A/T)AGAA-3' | ~200 bp |
|  | **a**2-R-deg | 5'-GA(C/T)AA(A/G)TATT(T/C)(A/G)AAATA(T/G/A)IT(T/C)TT-3' |  |

*For *C. metapsilosis*, primers designed by Pryszcz et al. [60] were used to amplify α1, α2, **a**1, and **a**2 genes; I, Inosine.
